# Effects of Head-Neck Positions on the Hand Grip Strength in Healthy Young Adults: A Cross-Sectional Study

**DOI:** 10.1155/2018/7384928

**Published:** 2018-07-25

**Authors:** Hamayun Zafar, Ahmad Alghadir, Shahnawaz Anwer

**Affiliations:** Rehabilitation Research Chair, College of Applied Medical Sciences, King Saud University, Riyadh, Saudi Arabia

## Abstract

**Background:**

Assessment of hand grip strength is vital for the evaluation of upper limb impairments and effective exercise prescription. Present study primarily aimed to investigate the effect of head-neck positions on the hand grip strength in healthy young adults. Secondarily, the present study compared hand grip strength between left versus right hand in different head-neck positions.

**Methods:**

Healthy young adults (age 19 – 30 year; n = 40) participated in this cross-sectional study. Hand grip strength was assessed in three head-neck positions (neutral, rotation left, and rotation right) using the standard adjustable Jamar hand dynamometer.

**Results:**

Hand grip strength in both sides (left and right) was greatest in the head-neck neutral position. Hand grip strength measured in head-neck left rotation position displayed the least strength in both sides. There was no significant difference noted between left and right side hand grip strength measured in head-neck neutral and right rotation positions. However, there was a significant difference noted between left and right side hand grip strength measured in head-neck left rotation positions. There was no significant effect of head-neck positions on hand grip strength noted in both sides.

**Conclusions:**

Hand grip strength was highest in the head-neck neutral position followed by head-neck rotation to the right. However, head-neck positions did not significantly affect hand grip strength in healthy young adults. Further studies assessing the hand grip strength in different neck positions in patients with neck pain and upper limb dysfunction may have significant implications for the assessment of hand grip strength.

## 1. Background

Assessment of hand grip strength is vital for the evaluation of upper limb impairments and effective exercise prescription [1, 2]. The hand is considered an important tool through which we manipulate and control our environment [3]. Previous studies recommended hand grip strength as a predictor of general health including heart disease [4], disability [5, 6], cognitive impairment [5, 6], cerebrovascular disease [5, 6], risk of future fracture [7, 8], and mortality [9, 10].

It was reported that body positions and upper limb positions can influence the measurement of hand grip strength [11, 12]. The tonic neck reflex (TNR) is a reflex phenomenon in which head positions affect limb muscle tone [13]. Although it may influence all four limbs, its effect is more on the upper limbs than the lower limbs [3]. The TNR has two components including a symmetrical tonic neck reflex (STNR) and an asymmetrical tonic neck reflex (ATNR) [14]. Previous studies indicated that, even in healthy infants and adults, persistent ATNR influences movements in specific states including motion stress and relaxation [15, 16].

Previously, Deutsch et al. [17] recommended the use of head-neck rotations during strengthening of upper extremity muscles to induce ATNR. Tokizane et al. [18] reported an influence of head-neck position on motor unit activity in infants, healthy adults, and neurologically impaired persons. In addition, Su et al. [19] reported an influence of shoulder and elbow positions on the hand grip strength in healthy adults. However, only few studies have investigated the influence of head-neck positions on the hand grip strength in healthy adults. Kumar et al. [3] reported that highest grip strength noted in head-neck rotation to left side. Similarly, Lee et al. [20] reported slightly increased grip strength measurement in head-neck rotation position compared to head-neck neutral in healthy adults. In contrast, Wong et al. [21] did not find an influence of head-neck rotation on the strength of the elbow flexors and extensors in healthy young women.

The present study aimed to investigate the effect of head-neck positions on the hand grip strength in healthy young adults. Secondarily, present study compared hand grip strength between left versus right hand in different head-neck positions.

## 2. Methods

A total of 40 male and female students between the ages of 19 and 30 years participated in this cross-sectional study. Participants were excluded if they had any history of sensory deficits in upper extremity, history of recent upper extremity surgery, recent upper extremity injury, and undergoing any type resistance training. Study procedures were explained verbally to each participant after getting a written informed consent. The study was approved by the ethics committee of our institution.

Hand grip strength was assessed in three head-neck positions (neutral, rotation left, and rotation right) using the standard adjustable Jamar hand dynamometer as reported in the previous published studies [3, 17] (J-Tech 12-0259 Commander, Grip Track Dynamometer, Midvale, UT). The participants were asked to maintain the neck rotation at the maximal possible range. In addition to head-neck positions, the American Society of Hand Therapists' standardized grip-strength testing procedure was followed [22]. The dynamometer was fixed at the second handle position for measurement of hand grip strength. The participants were instructed to squeeze the handle of the dynamometer maximally and maintain it for 5 seconds. Average of the three measurements was recorded for the analysis purpose. A rest of one minute and five minutes was provided between three measurements and three different head-neck positions, respectively. Three head-neck positions were randomized to minimize fatigue effect.

All statistical tests were done using the SPSS (Version 22. IBM Inc., Chicago, IL). Descriptive statistics including proportion, mean, standard deviation, and 95% confidence interval were presented. After testing normality of data, a repeated-measures analysis of variance (parametric tests) was used to compare hand grip strength in different head-neck positions.* t*-test was used to compare hand grip strength between left and right side in different head-neck positions. The results were considered statistically significant if p value was less than 0.05.

## 3. Results

**Figure 1:**
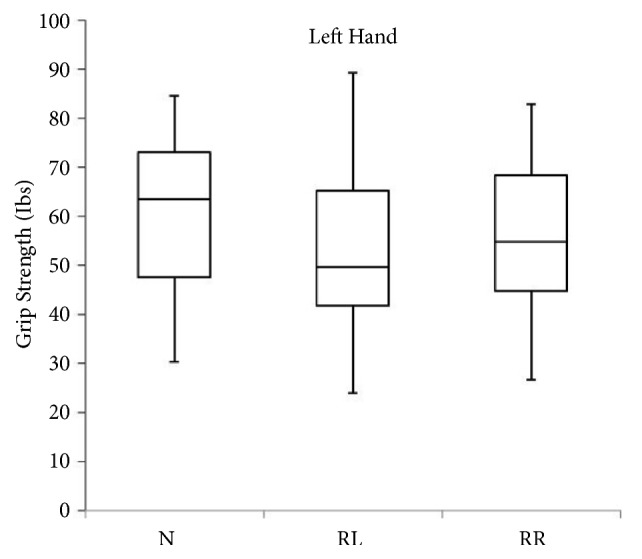
Box plot showing the distribution of hand grip strength in the left side measured in three neck positions (N, neutral; RL, rotation to left; RR, rotation to right).

**Figure 2:**
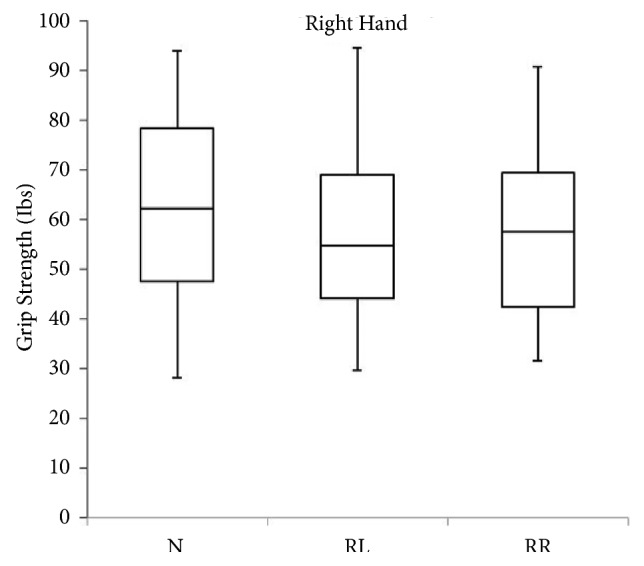
Box plot showing the distribution of hand grip strength in the right side measured in three neck positions (N, neutral; RL, rotation to left; RR, rotation to right).

A total of 40 subjects between the ages of 19 and 30 years participated in this study.  Table 1 gives the participants characteristics.  Table 2 presents the comparison between left and right hand grip strength in different head-neck positions. Hand grip strength in both sides (left and right) was greatest in the head-neck neutral position. In addition, hand grip strength measured in head-neck left rotation position displayed least strength in both sides. There was no significant difference noted between left and right side hand grip strength measured in head-neck neutral and right rotation positions (p > 0.05). However, there was a significant difference noted between left and right side hand grip strength measured in head-neck left rotation positions (p = 0.023).  Table 3 presents the comparison of hand grip strength in different head-neck positions using a repeated-measures analysis of variance. There was no significant effect of head-neck positions on hand grip strength noted in both sides (p > 0.05). Box plot showing the distribution of hand grip strength in the left and right side measured in three neck positions is presented in Figures 1 and 2.

## 4. Discussion

The primary aim of the present study was to investigate the effect of head-neck positions on the hand grip strength in healthy young adults. Secondarily, it compared hand grip strength between left versus right hand in different head-neck positions. The results of present study indicated that the measurement of hand grip strength in the head-neck neutral position yields greatest strength in both sides (left and right). However, there was statistically insignificant effect of head-neck positions on hand grip strength noted in both sides.

In contrast, Kumar et al. [3] reported highest grip strength measurement in head-neck rotation to left side. Similarly, Lee et al. [20] reported slightly better grip strength measurement in head-neck rotation position compared to head-neck neutral in healthy adults. However, Wong et al. [21] did not find any effect of head-neck rotation on the strength of the elbow flexors and extensors in healthy young women. There were some methodological differences between Lee et al.'s study and the present study. In Lee et al.'s [20] study, the Kinesio tape was applied to the flexor muscle of the hand during the measurement of hand grip strength. This could be one of the reasons for different results. As per proprietor of the Kinesio tape, it improves muscle function by strengthening weak muscles [23, 24]. In addition, Kinesio tape could cause physiological changes in the muscle and myofascial functions due to increase in the blood and lymph circulation at the taping area [23, 25]. Moreover, it is also suggested that the application of Kinesio tape may stimulate cutaneous mechanoreceptors and, therefore, Kinesio tape might improve muscle excitability [25, 26]. In the Kumar et al. [3] study, the participants were in the age group of 17-25 years; however, in the present study, the participant age was between 19 and 30 years. This might be one of the reasons for different results.

In this study, head-neck neutral and right rotation positions did not influence measurement of hand grip strength between left and right side. However, hand grip strength measured in head-neck left rotation position was significantly higher in the right side compared to the left side. This significant difference in grip strength (mean value of right is 56.5 compared to left 53.5 lbs) may be due to ATNR. Similarly the ATNR could have influenced greater mean value for left grip strength during neck rotation to right; but this possibly may not have occurred because the samples are comprised majorly (80%) of right handed dominant volunteers. There could be another reason for such result; that is, the study conducted includes both genders grouped together with majorly 65% males and 35% females. Deutsch et al. [17] conclude that the influence of the tonic neck reflex may be elicited more easily in female volunteers than in males, which is primarily due to the reduced limb strength in females. A comparison between both the genders would have been more effective in reducing this biased result. In addition, hand grip strength was higher in head-neck right rotation position compared to left rotation in both hands. The mechanism of TNR is thought to come into play for this finding. Past studies showed that, even in healthy infants and adults, presence of ATNR could influences movements in particular states including movement and relaxation [15, 16].

This study is limited to only young adults. Therefore, similar study with older adults is warranted. In addition, further studies assessing the hand grip strength in different neck positions in patients with neck pain and upper limb dysfunction such as shoulder pain, elbow pain, and wrist pain may have significant implications for the assessment of hand grip strength.

## 5. Conclusions 

The hand grip strength was highest in the head-neck neutral position followed by head-neck rotation to the right. However, head-neck positions did not significantly affect hand grip strength in healthy young adults.

## Figures and Tables

**Table 1:** Participants characteristics.

Gender, No. (%)	
Male	26 (65)
Female	14 (35)

Age, years	
Mean (SD)	25.60 (3.93)
Range	19 – 30

Body mass index, Kg./m^2^	
Mean (SD)	25.63 (4.43)
Range	18.14 – 34.11

Hand dominance, No. (%)	
Right	32 (80)
Left	8 (20)

**Table 2:** Comparison between left and right hand grip strength in different head-neck positions.

	Hand grip strength (lbs), Left			Hand grip strength (lbs), Right			*∗*P-value
	Mean	SD	95% CI	Mean	SD	95% CI	
N	61.1	15.3	56.2 – 65.9	62.6	17.4	57.3 – 67.8	.251
RL	53.5	15.6	48.7 – 58.4	56.5	17.1	51.3 – 61.8	.023*∗*
RR	55.6	15.7	50.7 – 60.4	57.8	15.9	52.6 – 63.1	.074

*∗*t-test (statistically significant if p < 0.05); N: neck in neutral position; RL: neck rotation left; RR: neck rotation right; CI: confidence interval.

**Table 3:** Comparison of hand grip strength in different head-neck positions using a repeated-measures analysis of variance.

**Tests of Inter-subjects Effects**					
**Dependent Variable: Hand Grip Strength (lbs), Left side**					

Source	Type III sum of squares	df	Mean square	F	*∗*P

Corrected model	1206.469	2	603.234	2.492	.087

Head-Neck Positions (N vs RL vs RR)	1206.469	2	603.234	2.492	.087

Error	28319.153	117	242.044		

**Dependent Variable: Hand Grip Strength (lbs), Right side**					

Corrected model	813.802	2	406.901	1.434	.242

Head-Neck Positions (N vs RL vs RR)	813.802	2	406.901	1.434	.242

Error	33187.739	117	283.656		

N: neck in neutral position; RL: neck rotation left; RR: neck rotation right.

## Data Availability

The data used to support the findings of this study are available from the corresponding author upon request.

## Abbreviations

TNR: Tonic neck reflex
ATNR: Asymmetrical tonic neck reflex
STNR: Symmetrical tonic neck reflex.
